# Genomic testing for rare disease diagnosis—where are we now, and where should we be heading? The reflections of a behavioural scientist

**DOI:** 10.1038/s41431-023-01439-0

**Published:** 2023-08-01

**Authors:** Celine Lewis

**Affiliations:** 1 Population, Policy and Practice Department, UCL Great Ormond Street Institute of Child Health, London, UK; 2 NHS North Thames Genomic Laboratory Hub, Great Ormond Street Hospital for Children NHS Foundation Trust, London, UK

**Keywords:** Psychology, Health services

As I write this, it is a few weeks since we celebrated 70 years since the discovery of the structure DNA which was published by Watson and Crick in the journal Nature on April 25th 1953. Since then, there have been enormous strides that have taken place in the world of genomics, including the publication of the human genome back in 2001, and the awe-inspiring advancements in high-throughput DNA sequencing technologies which have revolutionised our ability to decipher the genome with unprecedented speed and accuracy. We live in a world today where a human genome can be sequenced, analysed and a diagnosis returned in a matter of hours [1], a feat which was unimaginable 70 years ago.

## Genomics in the United Kingdom

Across the globe, from Australia to Brazil, China to Turkey, national genomic medicine initiatives are underway. However, a variety of approaches are being utilised and local healthcare contexts vary [2]. One area where genomic medicine has been particularly transformative is through improved diagnoses in the field of rare diseases. The Deciphering Developmental Disorders (DDD) study, which recruited more than 13,500 families across the United Kingdom (UK) and Ireland with severe, difficult-to-diagnose developmental disorders, was able to provide a diagnosis using genomic analysis for approximately 41% of probands in which standard, phenotypically driven diagnostic approaches had failed [3]. Similarly, the 100,000 Genomes Project, a world-leading initiative set up in England in 2015 with the explicit aim of embedding genomic medicine into clinical care, is showing the remarkable benefits of whole genome sequencing with diagnostic yields for intellectual disability, hearing disorders, and vision disorders ranging from 40 to 55% and with an overall diagnostic rate of 25% [4].

Building on the learnings from the 100,000 Genomes Project, 2018 saw the launch of the nationally commissioned Genomic Medicine Service (GMS) in England, which offers access to comprehensive and equitable genomic testing including whole genome sequencing across the country. The vision is that genomic testing will be accessible to all as part of routine care in the National Health Service (NHS) through embedding genomics from primary and community care through to specialist and tertiary care [5]. This is a new approach to genomic medicine which is clinically and scientifically led and responsive to innovation, new technologies and data-led insights. As such, research and innovation are key pillars of the new service. I would argue that this includes research as delivered by behavioural scientists like myself, using an organised, systematic and theory-informed approach to gain insights into stakeholder experiences and attitudes, decision-making processes, communication strategies and outcomes. Understanding of these processes, perspectives, social determinants and inter-personal mechanisms is key to ensuring that genomic advances are translated into patient and public benefits.

## What do we know about the benefits of genomic medicine for rare disease patients?

Much work has been done by behavioural scientists over recent years to understand patients’ and parents’ motivations for undergoing genomic testing. Findings highlight patients’ and parents’ desire: for a diagnosis to access treatments, for access to clinical trials and/or disease-specific screening; to receive a clear prognosis and information about recurrence risk; to understand the aetiology of the condition and receive a reason “why” it occurred; to gain relief from guilt, for example, that it was not caused by something the mother did during her pregnancy (a concern I frequently come across); to gain legitimacy for the patient’s behaviour and/or appearance; and to enable them the opportunity to connect with others through support groups and social media. We also know why some may have hesitancies or decline the offer of such testing, including lack of perceived benefits from a diagnosis; concerns around data access and confidentiality; and concerns around the psychological and emotional impact, particularly if the results might reveal unwanted or unexpected information including variants of uncertain significance or secondary findings.

Evidence around the clinical utility of genomic testing is also beginning to emerge and includes changes in clinical management and amended treatment plans. Rapid genomic testing for critically ill children in particular has shown to be transformative through the avoidance of unnecessary investigations, interventions and surgical procedures and facilitating access to precision medicines. Where a life-limiting condition has been diagnosed through rapid testing it has enabled direct access to palliative care, thus reducing unnecessary suffering. The evidence around the psychological outcomes of genomic testing for patients and parents is less clear-cut. A recent exploratory meta-analysis [6] found that overall, receiving results (including diagnostic and ‘no-findings’ results) did not lead to negative psychological effects. However, the story may be more complex, particular for paediatric populations where parents have been found to experience a range of emotions including relief, fear, disappointment, loss of hope and frustration, particularly if the diagnosis is so rare that very little information exists about it. However, this research is still in its infancy, and further research is essential to gain a more nuanced and complete understanding of the psychosocial and behavioural impact.

## A call to action

Research, and in particular behavioural science research which aims to better understand human psychology and behaviour, has a key role to play in ensuring that that the processes and outcomes of genomic testing are well established so that evidence-based recommendations can be made which influence policy and practice, maximising patient benefit and reducing potential harms. However, currently, I would argue that the field is hampered in the following ways.

## Diversity and inclusion

There is a lack of diversity amongst study participants with the vast majority of behavioural science research including participants from White, well-educated backgrounds. We need to be better at recruiting participants from Black and Asian communities as well as other under-represented groups in genomics research. One way to do this is by using strategies that have been shown to be effective, such as the use of trusted advocates, peer researchers and the involvement of local communities to improve the generalisability of the findings and to reduce the risk that genomic testing only caters for a subset of the population.

We are also still reliant on a model whereby we conduct research ‘on’ patients rather than ‘with’ patients. Democratising research and enabling patients, families and patient organisations to inform, influence and actively collaborate in research is vital. Working in partnership with those people who have ‘real-life’ experience relevant to genomics research, ensures that the research being conducted is not only fit for purpose but also better, relevant, acceptable and appropriate.

## Gaps in our understanding

Much of the research to date has focused on the immediate impact of receiving genomic test results, yet we know many of the benefits of a diagnosis are only established in the longer-term. Research is therefore needed to understand these longer-term impacts including clinical, behavioural and psychosocial outcomes. This includes, amongst other things, disease progression, reproductive decision-making, clinical management, patient/parent empowerment, family dynamics, patient/parent health-related quality of life and impact on “lifestyle” behaviours. Moreover, to date, research has tended to focus on those that receive a diagnostic result. Further research on those who do not receive a diagnostic result—which currently comprises the majority of patients—as well as those that receive a variant of uncertain significance (VUS) is therefore essential. This could include, for example, qualitative interviews with patients and parents to explore the lived experience, ethnographic observations to examine the communication that occurs (this would be particular interesting for VUSs), and quantitative surveys to assess psychological impact at scale.

Further research on the attitudes of those who decline genomic testing is also important to provide a more complete picture around the acceptability and accessibility of genomic medicine. The data shows that uptake of genomic testing amongst minority ethnic groups is lower than one might anticipate. These groups have legitimate concerns around genomic testing due to factors including historical and current experiences of discrimination, disparities in healthcare and cultural considerations. There needs to be better understanding of these concerns to ensure culturally competent genetic counselling and foster trust between genetic services and communities.

Finally, there is a lack of standardisation in terms of study design, in particular the measures and questions used (in both qualitative and quantitative studies) to examine patient/parents’ experiences, attitudes and outcomes. Moreover, whilst work is developing in this area, further development of validated scales is important, for example those that focus on rapid genomic testing in intensive care or newborn genome screening.

## A joined-up approach

In order to gain a rich, comprehensive and holistic understanding around the complex interrelationships relating to the impact, value, cost, benefits, risks, challenges and experiences, we need to ensure multi-disciplinary teams of researchers including behavioural scientists, implementation scientists, health economists, ethicists and data scientists are working collaboratively on large-scale, national projects. This will require foresight from funders and allocation of sufficient funds and resources. Research also needs to be embedded at all stages as we integrate new genomic technologies and programmes into our health services—this includes the planning stage, early pilots, roll-out and beyond.

Finally, and perhaps most importantly, it is becoming increasingly hard from a practical and logistical perspective, to conduct high-quality research. This is not only because of lack of time and funding for those people required to support the research process (I’m thinking about front-line staff who identify research participants, support recruitment, disseminate surveys etc), but also because the process for setting up studies (in particular when it involves recruitment through multiple sites which is common when working in rare disease) is long and arduous. In England, the NIHR Clinical Research Network supports research by meeting the costs of additional staff, facilities, equipment and support services. However, in my experience, there is still a gap in terms of what is currently available and what is actually needed. If we want to embed research at the heart of healthcare, we need to find ways to simplify and streamline this process, ensure sufficient funding for front-line research delivery staff, and embed a culture of research within healthcare. Regarding this last point, one way we, as researchers, could support this, is by ensuring we feed back the results of our research to those on the front-line, for example at departmental team meetings, to highlight the value of the research and also to foster a collaborative ‘team spirit’ with all involved in the research process.

## The time is now

There is justified excitement about the benefits of genomic medicine, not only in the world of rare diseases, but also in precision medicine, newborn screening, pharmacogenomics and predictive testing to name a few. At the same time, the ethical, legal and social ramifications of this technology are clear. All of us working in this immensely rewarding field want to maximise the benefits for patients and minimise harms. With foresight and planning, and ensuring that the behavioural and social science disciplines are at the heart of the genomics revolution, I believe this can be achieved.
